# Children with dyslexia show cortical hyperactivation in response to increasing literacy processing demands

**DOI:** 10.3389/fpsyg.2014.01491

**Published:** 2014-12-22

**Authors:** Frøydis Morken, Turid Helland, Kenneth Hugdahl, Karsten Specht

**Affiliations:** ^1^Department of Biological and Medical Psychology, University of BergenBergen, Norway; ^2^Department of Teacher Training and Pedagogy, UiT The Arctic University of NorwayTromsø, Norway; ^3^Division of Psychiatry, Haukeland University HospitalBergen, Norway; ^4^Department of Radiology, Haukeland University HospitalBergen, Norway; ^5^Department of Biomedicine, KG Jebsen Center for Neuropsychiatric Disorders, University of BergenBergen, Norway; ^6^Department of Clinical Engineering, Haukeland University HospitalBergen, Norway

**Keywords:** dyslexia, processing demands, orthography, compensatory, attention, sentence processing

## Abstract

This fMRI study aimed to examine how differences in literacy processing demands may affect cortical activation patterns in 11- to 12-year-old children with dyslexia as compared to children with typical reading skills. Eleven children with and 18 without dyslexia were assessed using a reading paradigm based on different stages of literacy development. In the analyses, six regions showed an interaction effect between group and condition in a factorial ANOVA. These regions were selected as regions of interest (ROI) for further analyses. Overall, the dyslexia group showed cortical hyperactivation compared to the typical group. The difference between the groups tended to increase with increasing processing demands. Differences in cortical activation were not reflected in in-scanner reading performance. The six regions further grouped into three patterns, which are discussed in terms of processing demands, compensatory mechanisms, orthography and contextual facilitation. We conclude that the observed hyperactivation is chiefly a result of compensatory activity, modulated by other factors.

## Introduction

This functional magnetic resonance imaging (fMRI) study investigated how changes in literacy processing demands affect cortical activation patterns in children with dyslexia compared to children with typical literacy development.

Dyslexia is a disorder of neurobiological origin (Lyon et al., 2003) that affects reading and writing acquisition and skills. In many cases, reading deficits may be more easily remediated or compensated than writing difficulties, which often persist into adult years (Berninger et al., 2008). Estimations of prevalence vary within the range of 5–17% (Gabrieli, 2009). The dominating account of dyslexia has been a phonological deficit affecting the ability to manage phonological input, compromising the acquisition of phoneme-grapheme correspondences and the ability to synthesize and analyze speech sound from print (Vellutino et al., 2004). This, in turn, hinders the process of extracting meaning from text (Shaywitz and Shaywitz, 2005). The exact nature of the phonological deficit remains under debate, but it is commonly thought to subsume three areas: phonological awareness, rapid automatized naming (RAN) and verbal short-term memory (STM). There is, however, mounting evidence that the latter two are rather independent predictors of dyslexia (Kibby, 2009; Kirby et al., 2010; Norton and Wolf, 2012).

Vellutino et al. (2004) define phonological awareness as the “conceptual understanding and explicit awareness that spoken words consist of individual speech sounds (phonemes) and combinations of speech sounds (syllables, onset-rime units),” and stress its importance for conceptually grasping the alphabetic principle (that letters correspond to sounds) and for learning how to map between phonemes and graphemes. Tasks requiring phonological awareness may involve smaller or larger phonological units, but generally ask for either a judgment (Do these two words start with the same sound?) or some manipulation (What word do you make if you remove /x/ from the word /xy/?) (Melby-Lervåg et al., 2012).

RAN is a measure of naming speed, or the ability to quickly retrieve phonological information from memory when faced with visual stimuli. It is an important component in general language processing speed, and a major contributor to the reading process (Denckla and Cutting, 1999; Georgiou et al., 2012; Norton and Wolf, 2012; Warmington and Hulme, 2012). The RAN deficit can be interpreted as reflecting either impoverished phonological representations despite intact semantic representations of words, or as an impairment in a more general timing mechanism, affecting the automatization of the reading process, and hence, reading fluency (Snowling, 2000). In their double-deficit hypothesis, Wolf and Bowers (1999) proposed that this deficit in naming speed, together with the deficit in phonological awareness, forms the core of developmental dyslexia.

However, reading also requires verbal STM to support the storage, manipulation and retrieval of verbal and written materials. In order to read, you have to be able to decode, and then remember what you have decoded in order to comprehend. Both processes put demands on verbal STM. Several studies have found evidence of a deficit in verbal STM in dyslexia (Jeffries and Everatt, 2004; Beneventi et al., 2009; Kibby, 2009; Trecy et al., 2013). Landerl et al. (2013), however, asserted that compared to phonological processing and RAN, verbal STM plays a relatively minor role.

Despite the dominance of the phonological deficit theory, research has shown that developmental dyslexia tends to be associated with deficits in a number of other neurocognitive functions like working memory capacity (Helland and Asbjørnsen, 2004; Smith-Spark and Fisk, 2007; Beneventi et al., 2010), visual skills (Valdois et al., 2004; Bosse et al., 2007; Vidyasagar and Pammer, 2010; Lobier et al., 2012), executive functions (Helland and Asbjørnsen, 2000; Beneventi et al., 2010), and long-term memory (Menghini et al., 2010). Dyslexia is not related to global IQ levels (Lyon et al., 2003; Tanaka et al., 2011), but has implications for pedagogical approaches and individual study techniques in and out of the classroom (MacKay, 1997; Gabrieli, 2009). Today, the multifactorial view of the disorder, which regards dyslexia as part of a continuum or dimensional space of literacy and general language aptitude (Bishop and Snowling, 2004; Pennington and Bishop, 2009), is gaining acceptance (Snowling and Hulme, 2012).

Most children progress through the process of reading acquisition in much the same way (Frith, 1985), a process that can be divided into three stages: The Pre-literacy stage, The Emergent Literacy stage, and the Literacy stage. The Pre-literacy stage is the period before formal reading instruction starts. This stage is characterized by the ability to recognize and “read” familiar logos belonging to for example a brand of toys or a favorite restaurant. The child would, however, not be able to read the same word in regular print by way of identifying the letters as the correct speech sounds and synthesizing them into a meaningful word. In the first phase of the Emergent Literacy stage the child is being taught and is gaining understanding of the grapheme-phoneme correspondence, and learns how to decode via analyzing and synthesizing the letters on the page and their corresponding speech sounds (alphabetic processing). As the child moves through the Emergent literacy stage he or she will increasingly be able to decode words without synthesizing individual sounds (orthographic processing). Finally, in the Literacy stage, reading has become automatized; the child will have knowledge of a substantial number of sight words, and can read connected text with relative ease.

Since reading is a skill that develops over time, the brain networks involved will be likely to change and develop with age (Shaywitz et al., 2007), reading level (Hoeft et al., 2007), and reading experience (Clark et al., 2014). This also holds for dyslexia as a developmental disorder of reading and writing, and we have to consider that it may present itself in different manners at different ages and skill levels (Helland and Morken, in review). Moreover, there are a number of mechanisms that may cause the networks to be different in typical and dyslexic readers: one is the neurobiological deficits that in themselves give rise to the disorder, a second is hyper- or hypoactivations associated with differences in reading level or reading experience and as such secondary deficits, a third is the possible engagement of compensatory mechanisms. These are all important issues when investigating dyslexia by way of imaging techniques, and we will discuss them in some detail below.

Dehaene (2009) suggested that efficient reading depends upon developing proper interconnectivity between visual areas [occipital regions and the ventral occipito-temporal region or the Visual Word Form Area which is thought to be specialized for the processing of letters and words (Cohen and Dehaene, 2004)] and general language processing areas, including a semantic network (inferior frontal region, anterior and middle temporal regions, anterior fusiform region and the angular gyrus) and a network for pronunciation and articulation (anterior insula, precentral region, superior temporal regions, and the supramarginal gyrus). Hence, dyslexia would arise from these interconnections not developing appropriately. Consequently, this model is in line with the multifactorial view of dyslexia, and does not provide a single unitary causal mechanism, but rather implies a complex network of possible causes. The two language networks identified within Dehaene's (2009) model correspond roughly to what has been termed the ventral and dorsal reading networks, respectively (Pugh et al., 2001; Sandak et al., 2004). However, Sandak et al. (2004) also postulated a third system, centered on inferior frontal regions and thought to be active in the decoding of new words during normal reading development. In Dehaene's (2009) model, these frontal regions are included in the two language networks. Sandak et al. (2004) held that the frontal and dorsal reading systems are especially important in beginning reading (before 10.5 years), with the ventral system increasingly engaged in skilled reading, and hence referred to as the “skill zone”. Within a stricter phonological deficit perspective this “skill zone” has been assumed to be secondary to the dorsal system in a causal explanation of dyslexia. For instance, McCandliss and Noble (2003) described a dysfunction of the left peri-Sylvian region, in itself causing the phonological deficit. In turn, this was thought to compromise the development and functional specialization of the Visual Word Form Area.

In accounting for causal mechanisms, interpreting the differences between typical and dyslexic readers is not straightforward. As pointed out, atypical patterns may have different causes. Hoeft et al. (2007) addressed this question, and proposed that regions showing hypoactivations in persons with dyslexia are core to the etiology of the disorder, whereas hyperactivations indicate regions of compensatory activity, i.e., regions showing a difference in activation due to level of reading skill, not as a result of dyslexia *per se*. Within such a view, hyperactivation could be seen as resulting from for example greater effort or greater attentional demands. Hence, one could also expect regions not belonging to the reading network *per se* to show differences between typical and dyslexic readers. However, the influence may also go the other way. A recent study by Clark et al. (2014) examining cortical thickness indicated that neuroanatomically the primary differences between subjects with and without dyslexia in the Pre-literacy stage lie within lower-level areas projecting to areas within the reading network. This indicates that the deficits within the reading network itself may be a result of a qualitative and quantitative difference in reading experience. This goes to show that the reading network is both influenced by and influences other regions of the brain, contributing to the very complex picture of the neurobiological signature of dyslexia. To add to the complexity, the same region may show inconsistent activation patterns across studies, making it difficult to draw firm conclusions.

The pattern of hyperactivation in certain regions accompanying hypoactivation in others has been found by a number of studies. Wimmer et al. (2010) noted that whereas there is a predominance of studies linking dyslexia to cortical hypoactivations in studies of English-speaking participants, hyperactivations are more often reported in studies of German-speakers. The authors linked this finding to differences in orthographic demands between the two languages. There are, still, a number of studies showing hyperactivation of mainly frontal regions in English-language studies. In a PET-study (positron emission tomography) of adult university students, Brunswick et al. (1999) found hypoactivation in subjects with dyslexia across a range of left hemisphere regions. However, they also reported dyslexic hyperactivation in a region of Broca's area and adjacent premotor areas when the task required reading aloud as opposed to silent reading. This hyperactivation was interpreted as compensatory activity supporting explicit reading and showing increased dependence upon sublexical processing. Along the same lines, Lehongre et al. (2011) discovered compensatory hyperactivation of the right auditory system in dyslexics during the presentation of auditory stimuli that typically activate the left more than the right auditory cortex. Shaywitz and Shaywitz (2005) reported compensatory mechanisms to be reflected in two ways: (1) in a demanding phonological task, older dyslexics showed greater activation than younger in left and right inferior frontal gyrus, and (2) there was a negative correlation between reading ability and brain activation in the right occipito-temporal region. Similarly, Shaywitz et al. (1998) suggested relative hypoactivation of posterior regions coupled with relative hyperactivation of frontal regions in dyslexics compared to non-impaired readers to constitute a neural signature for dyslexia. This frontal region of hyperactivation corresponds to the frontal network postulated by Sandak et al. (2004). Richlan et al. (2009) reported a meta-study of 17 different fMRI and PET studies from a variety of orthographies; shallow (Italian), semi-shallow (German and Swedish), and deep (English and French). In their most conservative analysis, they found hypoactivation of a large area of the left hemisphere (extending from dorsal inferior parietal to ventral occipitotemporal regions and into middle temporal and inferior frontal areas) accompanied by hyperactivation in areas of both the left (anterior insula, primary motor cortex, caudate nuclei, lingual gyrus, and thalamus) and right hemisphere (medial frontal cortex). In line with a number of other studies, the authors generally interpreted hypoactivations as reflecting a deficit in the functions subserved by the areas in question. The hyperactivations, mainly located in frontal and motor areas, on the other hand, were again interpreted as reflecting compensatory mechanisms (for example subvocal articulation) and increased effort. It was especially noted that the vast majority of studies contributing to the findings of hyperactivation were from German-language samples.

Hadzibeganovic et al. (2010) cautioned that orthography does have consequences for the processing demands of reading in different languages, which would imply that one should exercise caution when comparing neuroimaging studies across languages. This is further supported by studies showing that the predictive value of core factors like phonological processing and RAN varies with orthography. In a recent study, Moll et al. (2014) found that across orthographies, RAN predicts reading fluency whereas phonological processing predicts more of reading accuracy and spelling. Still, these predictive patterns were stronger in English, with its complex orthography, than in languages with more transparent orthographies. Also, as noted by Wimmer et al. (2010) the reading accuracy problem displayed by the majority of children with dyslexia in the English language sphere is much less obvious in more transparent orthographies. In these languages reading accuracy generally reaches ceiling early in the reading acquisition process, whereas problems in reading speed may persist for a longer period. Altogether, this supports that even though the stages of literacy acquisition are highly predictable, the process of literacy acquisition is influenced by the phonology and orthography of the language in question. More specifically, the relation between phonology and orthography is likely to affect the type of cognitive demands in force at the different literacy stages, and hence, what is required to achieve skilled reading.

Although dyslexia has most often been explained in terms of language and language related cortical areas there is some evidence that attention is also an integral part of the reading process, despite earlier consensus that skilled reading is a fully automatized process independent of attentional mechanisms (Shaywitz and Shaywitz, 2008). This idea is also reinforced by the finding that comorbidity of dyslexia and attention-deficit hyperactivity disorder (ADHD) is frequently observed (Willcutt and Pennington, 2000; Willcutt et al., 2000). Varvara et al. (2014) found that both auditory and visual-spatial attention explained variance in reading skill in their sample of children with and without reading deficits. Furthermore, Beidas et al. (2013) found that attention, along with naming speed and visual working memory contributed to reading measures after controlling for phonological skills. They suggested that linguistic information requires the majority of the limited attentional resources available to the dyslexic reader in order to achieve accurate decoding, at the expense of fluency. These findings may indicate relatively higher attentional demands in dyslexics than in typical readers.

In a previous study, we examined an overlapping sample of children from the same project as this study, but focused on younger children at risk of dyslexia (Specht et al., 2009). We found that 6-year-old children defined as at-risk showed different brain activation patterns compared to age-matched control children during alphabetic and orthographic processing. More specifically, we found that the groups differed in activation in the left angular gyrus, and in an occipitotemporal activation network related to alphabetic and orthographic processing, respectively. Importantly, the children were in the Pre-literacy stage, and had not started formal reading instruction in school. Still, the study showed that faced with a reading task, the two groups displayed different cortical activation. We now report a study that used the same stimulus set-up, with one condition added, that was used in the study by Specht et al. (2009), but this time applied to an overlapping group of older children (11–12 years). Previous studies have reported changes in the reading network related to age (Schlaggar et al., 2002; Turkeltaub et al., 2003; Shaywitz et al., 2007) and to literacy acquisition (Carreiras et al., 2009; Dehaene et al., 2010). Thus, looking at an older age group at a different point in literacy development is in itself interesting. At this point all participants had functional reading skills, albeit poorer for the children with dyslexia. As mentioned, the sample used in the present study was overlapping, but not identical, to the one in our previous study. Also, by now a dyslexia group was defined. The aim of the study was to investigate differences in cortical activation patterns in a group of children with dyslexia compared to a group of children with typical reading skills using fMRI, and further to examine whether this interacted with demands for literacy processing. The children were now in the Literacy stage, and to further add to the processing demands and ensure that the paradigm was appropriate, a third level was added to the original paradigm: the ability to process full sentences.

Few studies have addressed the relation between word and sentence processing. Rimrodt et al. (2009) conducted a study of sentence comprehension in a similar, but wider age-group. They investigated children between 9 and 14 years old with and without reading disability, finding increased activity in areas of linguistic processing (left middle/superior temporal gyri), attention and response selection (largely right hemisphere regions) in sentence reading when controlling for word recognition. The sentence reading task included sentences that were either meaningful or non-meaningful. The differences between the groups were mainly driven by the non-meaningful sentences. In a different study, Constable et al. (2004) looked at sentence complexity as well as input modality (auditory vs. print) effects in a group of adults with typical literacy skills. They found a group of regions in the left hemisphere with up-regulation of activity in more complex sentences independent of input modality. The authors suggested that these regions make up a cortical system with a central function in sentence parsing. Finally, Cutting et al. (2006) examined sentence comprehension in a group of unimpaired adult readers, while controlling for single word reading and verbal (words) STM. They found that sentence processing was associated with bilateral, though lateralized toward the left hemisphere, activation of the temporal cortex, as well as the occipital lobe and middle frontal gyri.

In line with previous research, we hypothesized that (1) there would be group differences in brain areas associated with language processing as well as compensatory activity, and (2) increasing processing demands from alphabetic to orthographic to sentence processing would enhance the differences between the groups. These hypotheses were largely confirmed by our study.

## Method

### Participants

The participants were recruited through The Bergen Longitudinal Dyslexia Study (Specht et al., 2009; Helland et al., 2011a; Morken and Helland, 2013; Clark et al., 2014). The original sample was 109 5-year-old children from four different communities in Western Norway, two urban and two rural. Norwegian has two official written norms (nynorsk and bokmål) that are, however, relatively similar. Both were represented in the sample. At the beginning of the project, a risk index questionnaire (RI-5) was distributed to parents and preschool teachers. The questionnaire was designed to identify children at risk of developmental dyslexia, based on factors shown by research to be associated with this disorder, such as heredity (Torppa et al., 2011), history of language development (Snowling et al., 2000), and development of motor skills (Nicolson et al., 2001) (please see Helland et al., 2011a for further details on the questionnaire). Twenty-six children (13 boys, 13 girls) were identified as at-risk based on the completed questionnaires. A control group was identified among the remaining children, matching the at-risk group on gender, age, and which preschool they attended (for further details on the selection process, please refer to Helland et al., 2011a). When the children were in the 6th grade, a follow-up study was conducted, of which the present study is part. For the fMRI-session 29 children (15 boys, 14 girls) agreed to participate.

Based on data collected in the follow-up, the children were divided into a dyslexia group (11 children, 5 boys, 6 girls) and a typical group (18 children, 10 boys, 8 girls). The dyslexia test battery consisted of four different literacy measures: non-word reading, real word reading, text reading, and spelling. Non-word reading, real word reading, and spelling were sub-tests from the test battery *Standardisert Test i Avkoding og Staving* (STAS) (Standardized Test of Decoding and Spelling) (Klinkenberg and Skaar, 2001). Carlsten Reading Test Grade 6 (Carlsten, 2002) was used to measure text reading (silent reading fluency and comprehension). This is a cloze test. The tests and scoring procedures are described in further detail in Helland et al. (2011a). *T*-tests showed significant differences between the groups in all four literacy measures; non-word reading (Dyslexia < Typical, *p* < 0.004, *d* = −1.304), real word reading (Dyslexia < Typical, *p* < 0.003, *d* = −1.313), spelling (Dyslexia < Typical, *p* < 0.003, *d* < −1.332) and text reading (Dyslexia < Typical, *p* < 0.0001, *d* = −2.05). The *t*-tests did not show significant group differences in age [mean age dyslexia = 11:10 (*SD* = 3.01 months), typical = 11:8 (*SD* = 2.96 months), *p* < 0.17] or in full-scale IQ [mean dyslexia = 100.2 (*SD* = 17.0), mean typical = 105.3 (*SD* = 12.1), *p* < 0.35] as measured with the Wechsler Preschool and Primary Scale of Intelligence (WPPSI) (Wechsler, 2002) at 5 years of age. All IQ scores were within normal range.

In Norway, very few students attend private schools. According to Statistics Norway (www.ssb.no), 97.4% of students attended public school per 2010. The few private schools that exist generally offer alternative pedagogical methodology. All schools in the present study were public. Furthermore, Norwegian society is rather egalitarian, homogenous, and has a large middle-class. Thus, socio-economic factors are of less concern compared to many other countries (Halvorsen and Stjernø, 2008).

Due to ethical considerations, the children received computer-based literacy training as part of the project. The training was administered as three intensive periods of 4–5 20 min sessions per week for 8 weeks, one period per year for the first 3 years of the project (age: ~5–7 years). The sessions were put together using evidence-based methods for targeted literacy intervention. Still, they were conducted in accordance with the current school curriculum, meaning that no direct reading or writing training was given for the first 2 years. The children were divided into two training types, top-down (going from text to phonemes) or bottom-up (going from phonemes to text) (for details about the training scheme, please refer to Helland et al., 2011b). A repeated measures ANOVA with the design 2 groups (Dyslexia, Typical) by 2 training types (bottom-up, top-down) did not yield any significant interaction effect, indicating that that the two training types had about the same effect on the literacy scores. The reasons for including these training sessions were twofold; (1) to give something back to the children as a compensation for the substantial amount of time they put into their participation, and (2) to provide the schools with updated competence as recognition for the work they put down for the project. The ethics committee would not have approved the project without the training periods, and as such this was a matter of going through with the research or not.

All parents filled out written informed-consent forms on behalf of their respective child. Both the Bergen Longitudinal Dyslexia Study and the follow-up study were approved by the Regional Committee for Research Ethics in Western Norway and by the Norwegian Social Science Data Services.

### Stimuli

**Figure 1:**
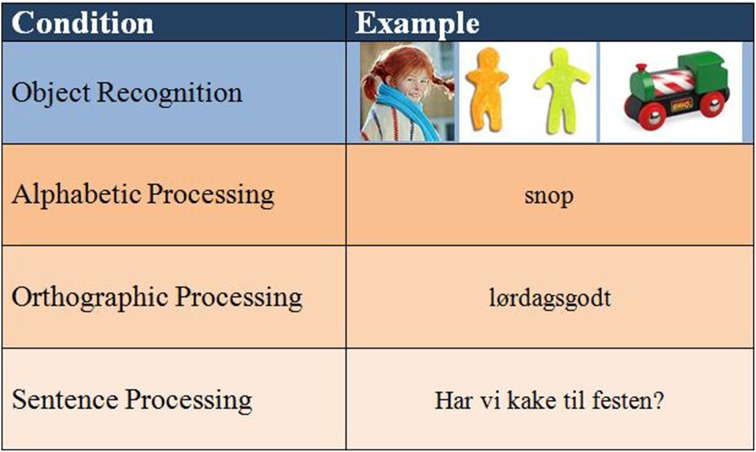
**Paradigm with stimulus examples**.

The paradigm was constructed in accordance with suggested stages of literacy development, and the corresponding reading processing strategies (alphabetic/orthographic) and also in accordance with a previous study done by our group (Specht et al., 2009). However, because the children in our study were already in the 6th grade, the original paradigm was slightly modified in two aspects. First, even those struggling with literacy were expected to have moved out of the Pre-literacy stage. Hence, stimuli related to this stage (“reading” of logos) were omitted, so as to not bore or fatigue the participants. Second, a level of sentence processing was added in order to further strain their reading capacity. The first level of stimuli was object recognition. The participants were presented with pictures of objects or characters. This condition is not directly related to literacy, and, as such, will not be further discussed here. There were three literacy conditions: The Emergent literacy stage was first represented by short words with regular spelling, allowing the use of an alphabetic strategy for decoding. This was considered the easiest condition. The second level also belonged to the Emergent literacy stage, but consisted of longer and more irregular words that would be difficult to decode via an alphabetic route, hence necessitating an orthographic reading strategy. Finally, the sentence level further increased processing demands, and represented the Literacy stage. The sentence stimuli were of varying length, and were either declarative, interrogative, or contained negations or inversions. The different conditions were designed to represent an increase in literacy processing demands from alphabetic to orthographic to sentence processing (see Figure 1 for an overview of the final paradigm with examples).

### FMRI procedure

Stimulus presentation, synchronization with trigger signals, received from the MR scanner, and recording of responses were managed by the E-prime software (v. 2.0: Psychology Software Tools, Pittsburg, PA: www.pstnet.com). Stimuli were presented visually in a block design using scanner-compatible goggles (www.nordicneurolab.com). The paradigm had four different conditions: object recognition, alphabetic processing (short words), orthographic processing (long words), and sentence processing. Only the three latter conditions (the literacy conditions) are reported and discussed further below. There was one run per condition, each consisting of four ON-blocks and five OFF-blocks. In each ON-block, four different stimuli were presented, belonging to one of four categories: “things to play with,” “things to see on TV,” “things to eat,” or “things to drink,” of which one was the target category. The order of the stimuli was randomized. The children were given the task of pressing a button on the response grips provided (www.nordicneurolab.com) in response to one of the categories. Hence, the task necessitated decoding followed by semantic judgment in all three literacy conditions. The literacy processing required to reach a decision did, however, differ between the conditions. The different task categories were randomized across the children, but each child had the same task across all conditions. The children were trained beforehand on a similar task outside the scanner to ensure that the task was understood. The stimuli used during this training session, were not identical to the ones used during image acquisition. Each stimulus was presented for 5 s with a 1 s blank screen between stimuli, giving ON-blocks of 24 s. OFF-blocks were of the same length, and consisted of a blank screen. All stimuli, including the sentences, fitted on one single line on the screen. The full MR-protocol lasted about 45 min per child.

### Image acquisition

Images were acquired on a General Electric Signa 3.0-T scanner equipped with 40 mT/m TwinSpeed gradients and Quiet Technology. A 3D T1-weighted Fast SPGR Sequence with188 sagittal slices (1 mm thickness) with a voxel size of 1.02 × 1.02 mm was used for the acquisition of anatomical data. To acquire BOLD (Blood Oxygenation Level Dependent) data, a total of 320 EPI-volumes were collected, 80 volumes per run, with the following parameter: *TR* = 3.0 s, *TE* = 30 ms, 1.72 × 1.72 mm in-plane voxels, 128 × 128 matrix, slice thickness 3.5 mm, 0.5 mm gap, and 35 axial slices. Each ON-block allowed the acquisition of eight full scans, and the same for each OFF-block. Each run was preceded by eight dummy scans in order to assure signal saturation of the cerebrospinal fluid facilitating steady state signal intensity.

### Behavioral data

Reading performance in the scanner was measured by registering the number of correct responses as well as average response times per condition. Due to technical issues behavioral data were not recorded for six participants (1 dyslexia, 5 typical). We have no reason to assume that the results of these participants would differ systematically from the remaining group.

### Data analyses

FMRI data were analyzed using the SPM8 software (Wellcome Department of Cognitive Neurology, http://www.fil.ion.ucl.ac.uk) executed in MATLAB (www.mathworks.com). Before data analyses, the images were pre-processed using realignment and unwarping procedures in SPM8. The realignment reports for each participant were inspected visually to ensure that the amount of movement was acceptable, i.e., less than 2 mm and 2°. The images were then normalized onto the MNI-standard space, defined by an EPI template in SPM8. Finally the data were smoothed, using an 8 mm Gaussian kernel.

The statistical analyses of the fMRI data included four steps. First, an individual fixed effects analysis was performed. Based on the general linear model framework, a design matrix was defined that contained the three conditions (alphabetic, orthographic, and sentence processing). Each block was modeled by its onset time and its duration of 24 s. The resulting onset vector was convolved with a hemodynamic response function (hrf), provided by SPM8. The data were filtered with a high-pass filter of 128 s. For each of the three conditions, contrasts were defined, representing the differences in BOLD signal between the ON and OFF blocks. The corresponding contrast images were subjected to second-level analyses. Second, based on these contrast images, a random effects one-sample *t*-test was estimated for each literacy condition and each group (dyslexia and typical) in order to identify areas of activation. These data are reported with a peak threshold of *p* < 0.001, and an extent threshold of *p* < 0.05 (FWE-corrected). Third, differential group effects were explored with a 2 (typical/dyslexia) × 3 (alphabetic/orthographic/sentences) factorial ANOVA. These data are reported at a significance level of *p* < 0.001 uncorrected with an extent threshold of at least 10 voxels per cluster. Finally, to examine the effects of increasing demand for literacy processing in more detail, the regions showing an interaction between group and condition went into a confirmatory regions of interest (ROI) analysis, again using a repeated-measures ANOVA with the design group (2) by condition (3). As input for this confirmatory *post-hoc* ANOVA served the individual beta-weights from the respective peak voxel. Fisher's least-significant-difference (LSD) test was used as a *post-hoc* test for significant differences between means, in addition to *t*-tests to further explore group differences. The reason for choosing the LSD test over a more conservative test like for example Tukey's HSD test, was to avoid the risk of Type-II error due to a relatively small N. The alpha-level for the ROI analyses was set at *p* < 0.05. For the repeated measures ANOVA, eta squared (η^2^) was used as a measure of effect-size. For the *t*-tests Cohen's *d* was used.

The behavioral data were analyzed with *t*-tests to check for performance differences between the two groups. Cohen's *d* was used to measure effect size.

## Results

### One-sample *t*-tests

**Figure 2:**
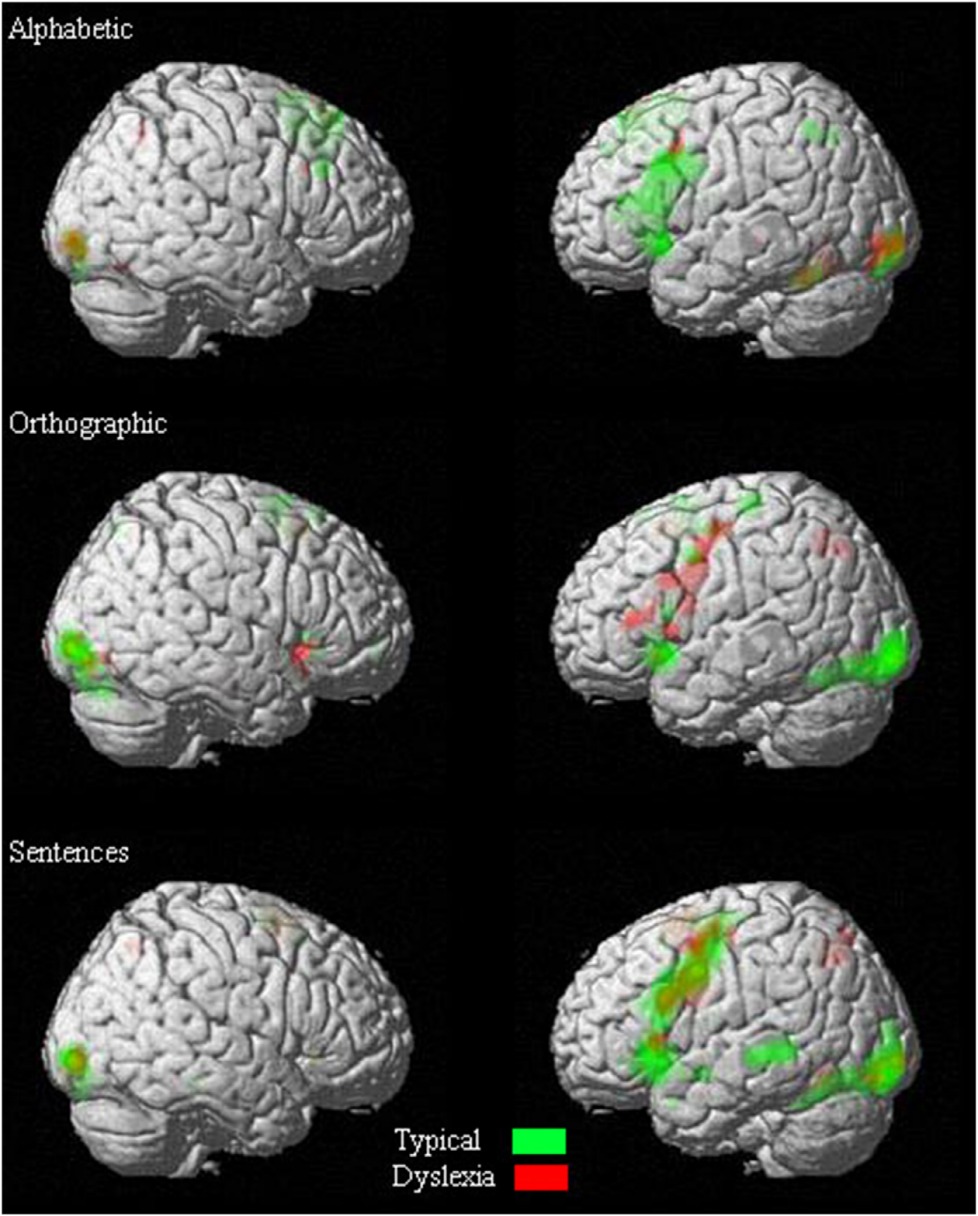
**Overlapping of activations in one-sample *t*-tests of each condition**.

One-sample *t*-tests were first done exploratively to identify areas of activation for each group separately. Figure 2 illustrates the degree of overlap in activation pattern between the two groups.

#### Alphabetic processing

The typical group showed significant activations bilaterally in the superior frontal gyrus, the middle cingulate, the lingual gyrus, inferior occipital gyrus, the supplementary motor area, and the cerebellum. Furthermore, they showed significant activation in the left inferior frontal gyrus, pre-central gyrus, the insula, middle occipital gyrus, caudate nucleus, and thalamus (see Table 1 for details).

**Table 1:** **Group results for significant activations per condition**.

**Anatomical localization**	***x***	***y***	***z***	**Side**	***p*-value (peak)**	***t*-value**	***p*-value (cluster)**	**Cluster size**
**ALPHABETIC PROCESSING**								
***Typical***								
Superior frontal gyrus, middle cingulate, supplemental motor area	0	26	50	L/R	0.001	8.15	0.001	1419
Inferior frontal gyrus, pre-central gyrus, insula	−48	18	32	L	0.001	7.55	0.001	2322
Lingual gyrus, inferior and middle occipital gyrus, cerebellum	−26	−92	−16	L	0.001	6.63	0.024	330
Lingual gyrus, inferior occipital gyrus, cerebellum	24	−90	−18	R	0.001	4.48	0.045	274
Caudate nucleus, thalamus	−10	10	22	L	0.001	5.80	0.016	364
***Dyslexia***								
Inferior occipital gyrus, lingual gyrus	32	−92	−8	R	0.001	9.31	0.015	262
Inferior and middle occipital gyrus, lingual gyrus	−34	−84	−16	L	0.001	4.31	0.002	383
Inferior temporal gyrus, fusiform gyrus	−48	−50	−22	L	0.001	4.17	0.028	224
**ORTHOGRAPHIC PROCESSING**								
***Typical***								
Inferior occipital gyrus, calcarine sulcus, fusiform gyrus, lingual gyrus, cerebellum	36	−92	−8	R	0.001	5.71	0.001	1055
Inferior and middle occipital gyrus, calcarine sulcus, lingual gyrus	−28	−94	−4	L	0.001	5.57	0.001	1248
Insula, orbitofrontal cortex, superior temporal pole	−52	12	−8	L	0.001	7.20	0.011	406
Supplemental motor area	2	6	66	L/R	0.001	4.59	0.004	503
***Dyslexia***								
Inferior frontal gyrus, pre-central gyrus	−44	14	30	L	0.001	9.64	0.001	651
Superior and inferior parietal sulcus	−24	−70	44	L	0.001	8.32	0.032	194
Lingual gyrus, calcarine sulcus, middle occipital gyrus	18	−92	−6	R	0.001	6.71	0.005	297
Insula, inferior frontal gyrus	−34	24	16	L	0.001	6.23	0.012	245
Insula, inferior frontal gyrus	30	20	−16	R	0.001	3.88	0.044	179
**SENTENCE PROCESSING**								
***Typical***								
Calcarine sulcus, lingual gyrus, inferior occipital gyrus	20	−96	−6	R	0.001	10.55	0.001	621
Inferior and middle frontal gyrus, pre- and post-central gyri, superior temporal pole	−48	24	−10	L	0.001	9.26	0.001	2910
Middle temporal gyrus	−54	−36	0	L	0.001	8.93	0.001	599
Inferior and middle occipital gyrus, calcarine sulcus, lingual gyrus, fusiform gyrus	−30	−92	2	L	0.001	8.57	0.001	1540
Thalamus	−6	−36	2	L	0.001	6.33	0.018	306
Supplemental motor area	−2	4	64	L	0.001	6.10	0.041	244
***Dyslexia***								
Precentral gyrus, inferior frontal gyrus	−40	0	36	L	0.001	9.98	0.001	1066
Supplemental motor area	−2	6	60	L/R	0.001	8.88	0.007	323
Lingual gyrus	−12	−90	−14	L	0.001	6.62	0.012	290
Lingual gyrus, inferior occipital gyrus	34	−92	−6	R	0.001	5.96	0.030	232

*Cluster size (number of voxels 2 × 2 × 2 mm), p-values for the peak voxel (uncorrected) and the cluster size (FWE-corrected), t-values, MNI coordinates of peak voxel and anatomical locations*.

The dyslexia group showed significant activations bilaterally in the inferior occipital gyrus and lingual gyrus. Moreover, they showed significant activation in the left middle occipital gyrus, inferior temporal gyrus, and fusiform gyrus (see Table 1 for details).

#### Orthographic processing

The analyses showed that the typical group had significant activations bilaterally in the inferior occipital gyrus, calcarine sulcus as well as in the lingual gyrus and the supplementary motor area. They also had significant activations in the right fusiform gyrus and cerebellum. Finally, there were significant activations in the left middle occipital gyrus, insula, inferior frontal gyrus, and superior temporal pole (see Table 1 for details).

The dyslexia group had significant activations bilaterally in the insula and inferior frontal gyrus. Furthermore, they showed significant activation in the left pre-central gyrus, superior and inferior parietal sulcus, and the right lingual gyrus, calcarine sulcus, and middle occipital gyrus (see Table 1 for details).

#### Sentence processing

For sentence processing, the typical group showed significant activations bilaterally in the calcarine sulcus, lingual gyrus, and the inferior occipital gyrus. Furthermore, they showed significant activation in the left inferior and middle frontal gyrus, pre- and post-central gyri, superior temporal pole, middle temporal gyrus, middle occipital gyrus, fusiform gyrus, thalamus, and supplementary motor area (see Table 1 for details).

For the dyslexia group, the analysis showed significant activations bilaterally in the supplementary motor area and the lingual gyrus. Also, there were significant activations in the right inferior occipital gyrus, left pre-central gyrus, and left inferior frontal gyrus (see Table 1 for details).

### Factorial ANOVA

The factorial ANOVA returned a main effect of group in the left calcarine sulcus, and in the right middle occipital gyrus, supplementary motor area, and inferior occipital gyrus (see Table 2 for details).

**Table 2:** **Results of factorial ANOVA**.

**Anatomical localization**	***x***	***y***	***z***	**Side**	***p*-value**	***F*-value**	**Cluster size**
**MAIN EFFECT OF GROUP**							
Middle occipital gyrus	32	−94	2	R	0.001	21.12	65
Calcarine sulcus	−20	−100	−4	L	0.001	18.13	20
Supplemental motor area	10	−8	70	R	0.001	15.06	20
Inferior occipital gyrus	40	−78	−6	R	0.001	14.85	18
**MAIN EFFECT OF CONDITION**							
Fusiform gyrus, lingual gyrus, inferior and middle occipital gyrus, calcarine sulcus, cerebellum	32	−54	−16	R	0.001	50.92	6173
Fusiform gyrus, lingual gyrus, cerebellum	−28	−72	−14	L	0.001	31.81	1214
Posterior and middle cingulate cortex	6	−24	26	L/R	0.001	23.59	1185
Supplemental motor area	−4	2	62	L	0.001	17.90	382
Pre- and post-central gyri, inferior frontal gyrus	−46	−4	58	L	0.001	17.84	2550
Superior parietal sulcus	−26	−56	50	L	0.001	15.25	514
Middle temporal gyrus	−50	−38	0	L	0.001	13.81	900
Angular gyrus	52	−58	38	R	0.001	13.04	522
**INTERACTION EFFECT: GROUP BY CONDITION**							
Superior frontal gyrus	14	22	46	R	0.001	8.65	107
Caudate nucleus	−12	4	18	L	0.001	8.00	53
Superior frontal gyrus	8	44	48	R	0.001	6.94	11
Middle cingulate cortex	−12	18	36	L	0.001	6.87	27
Middle frontal gyrus	32	24	48	R	0.001	6.85	11
Pre-supplemental motor area	−8	4	60	L	0.001	6.84	19

*Cluster size (number of voxels 2 × 2 × 2 mm), p-values, F-values, MNI coordinates of peak voxel and anatomical locations*.

Furthermore, there was a main effect of condition bilaterally in the fusiform gyrus, lingual gyrus, posterior and middle cingulate, and cerebellum. This effect was also significant in the left supplementary motor area, pre- and post-central gyri, inferior frontal gyrus, superior parietal sulcus and middle temporal gyrus, and in the right angular gyrus (see Table 2 for details).

Finally, there was a significant interaction effect between group and condition in the following six areas: Right superior frontal gyrus (R-SFG-post), left caudate nucleus (L-NC), right superior frontal gyrus somewhat more anterior than the first region (R-SFG-ant), left middle cingulate (L-MCC), right middle frontal gyrus (R-MFG), and the left pre-supplementary motor area (L-Pre-SMA) (see Table 2 for details).

ROI analyses were used in place of regular *post-hoc* testing to explain the nature of the interaction effect.

### ROI analyses

**Figure 3:**
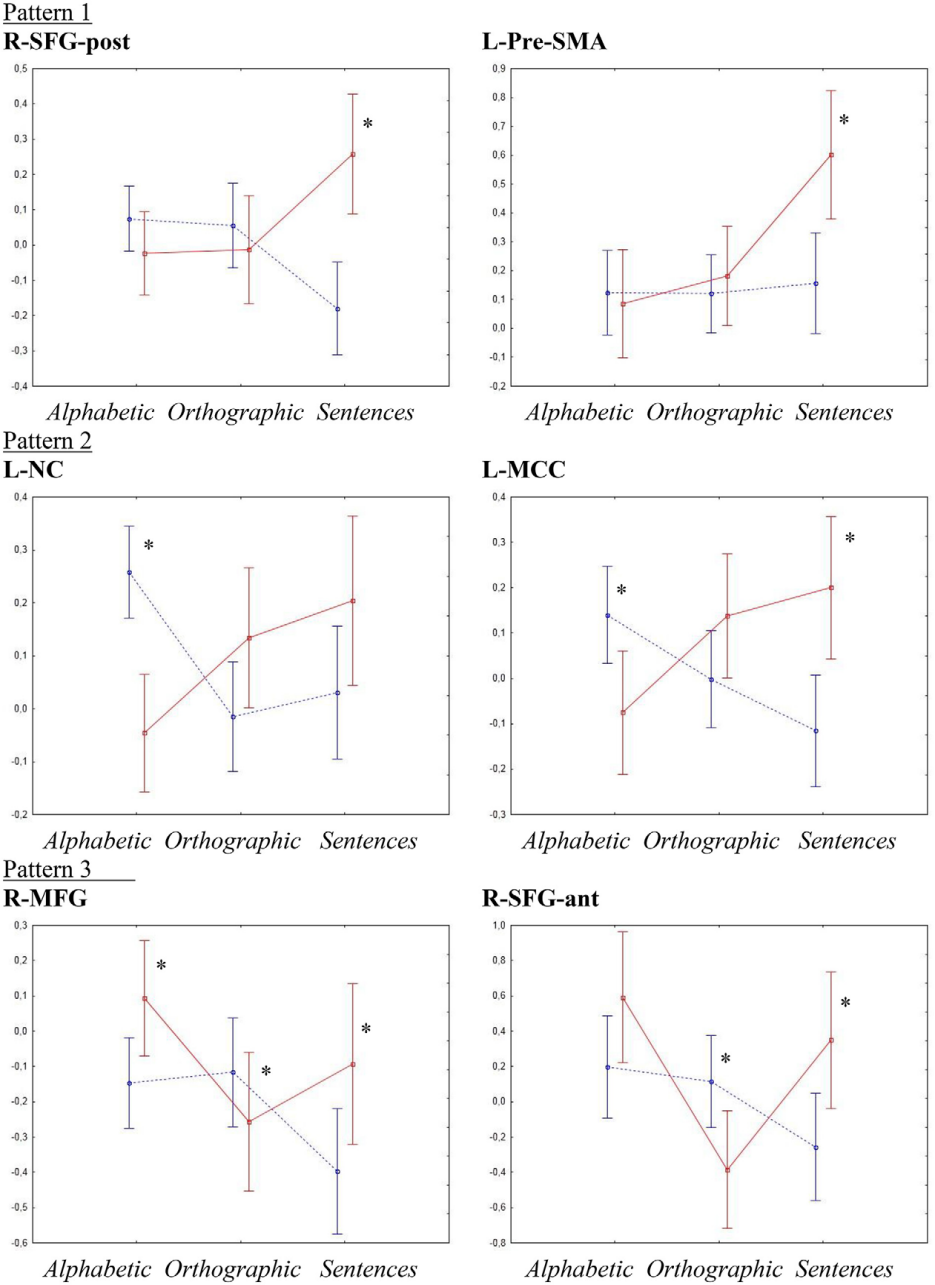
**ROI analyses interaction effects**. Y-axes show activation levels. Significant t-tests are marked by ^*^. The typical group is shown as a dotted blue line and the dyslexia group is shown as a solid red line.

To investigate the effects of increases in literacy processing demand, ROI analyses with repeated measures ANOVA followed by Fisher's LSD and *t*-tests were performed for the six regions showing an interaction effect of group by condition on the factorial ANOVA, indicating a difference in the way the typical and the dyslexia group handled changes in processing demand. Figure 3 shows an overview of the activation patterns in the different ROI.

#### Right superior frontal gyrus (posterior site)

R-SFG-post showed, besides the significant interaction effect of group by condition [*p* < 0.001, *F*_(2, 54)_ = 8.91, η^2^ = 0.24], a significant main effect of group [*p* < 0.03, *F*_(1, 27)_ = 4.99, η^2^ = 0.032]. Fisher's LSD showed that the group effect was due to activations in the dyslexia group being higher than in the typical group (*p* < 0.03), whereas the interaction effect was due to a higher activation in the sentence condition for the dyslexia group than all other activations (*p* < 0.05) and activations of the typical group in the sentence condition being lower than all conditions for the typical group and the sentence condition for the dyslexia group (*p* < 0.01). *T*-tests showed a significant difference between the groups only in the sentence condition (Typical < Dyslexia, *p* < 0.001, *d* = 1.547).

#### Left caudate nucleus

For the L-NC, the ANOVA confirmed the significant interaction effect of group by condition [*p* < 0.001, *F*_(2, 54)_ = 8.56, η^2^ = 0.24]. *Post-hoc* testing indicated that this was due to the activation for the typical group in the alphabetic condition being higher than both other conditions for the same group and the alphabetic condition for the dyslexia group (*p* < 0.01), as well as activations in the sentence condition for the dyslexia group being higher than the orthographic and sentence conditions for the typical group and the alphabetic condition for the dyslexia group. The *t*-tests corroborated these results, showing a significant difference between the groups in the alphabetic condition (Dyslexia < Typical, *p* < 0.0001, *d* = −1.783).

#### Right superior frontal gyrus (anterior site)

In the R-SFG-ant, besides the significant interaction effect of group by condition [*p* < 0.002, *F*_(2, 54)_ = 6.71, η^2^ = 0.17], a significant main effect of condition [*p* < 0.006, *F*_(2, 54)_ = 5.66, η^2^ = 0.14] was detected. A Fisher LSD test showed that the main effect of condition was due to activations for the alphabetic conditions being higher than activations for the orthographic condition (*p* < 0.02), while the interaction effect was mainly due to activations in the orthographic condition for the dyslexia group being significantly lower than all other activations except the sentence condition for the typical group (*p* < 0.03). *T*-tests showed significant differences between the groups in the orthographic condition (Dyslexia < Typical, *p* < 0.02, *d* = −0.931) and in the sentence condition (Typical < Dyslexia, *p* < 0.02, *d* = 0.936).

#### Left middle cingulate cortex

The ANOVA confirmed the significant interaction effect of group by condition in the L-MCC [*p* < 0.001, *F*_(2, 54)_ = 8.04, η^2^ = 0.22]. *Post-hoc* testing showed that this was due to activations for the typical group in the sentence condition being lower than both the alphabetic condition for the typical group and the orthographic and sentence conditions for the dyslexia group (*p* < 0.01). Furthermore, activations for the dyslexia group in the sentence condition was higher than the alphabetic condition for the same group and the orthographic and sentence conditions for the typical group (*p* < 0.02). *T*-tests showed significant differences between the groups in the alphabetic condition (Dyslexia < Typical, *p* < 0.017, *d* = 0.948) and the sentence condition (Typical < Dyslexia, *p* < 0.003, *d* = 1.239).

#### Right middle frontal gyrus

In the R-MFG, there was a significant main effect of condition [*p* < 0.013, *F*_(2, 54)_ = 4.7, η^2^ = 0.11], as well as the significant interaction effect of group by condition [*p* < 0.003, *F*_(2,54)_ = 6.62, η^2^ = 0.17]. Fisher LSD showed that the main effect of condition was due to higher activations overall in the alphabetic condition than in the sentence condition (*p* < 0.01). The interaction effect was mainly due to activations for the sentence condition in the typical group being lower than all others except the orthographic condition for the dyslexia group (*p* < 0.02), and activations for the alphabetic condition for the dyslexia group being higher than activations in the sentence condition for the typical group and the orthographic condition for the dyslexia group (*p* < 0.01). *T*-tests showed significant between-groups differences in all three conditions; alphabetic (Typical < Dyslexia, *p* < 0.02, *d* = 0.898), orthographic (Dyslexia < Typical, *p* < 0.05, *d* = −0.596), and sentences (Typical < Dyslexia, *p* < 0.04, *d* = 0.810).

#### Left pre-supplementary motor area

Similar to the voxel-wise SPM analysis, the ANOVA for L-Pre-SMA showed significant main effects of group [*p* < 0.05, *F*_(1, 27)_ = 4.29, η^2^ = 0.07] and condition [*p* < 0.003, *F*_(2, 54)_ = 6.5, η^2^ = 0.26] as well as an interaction effect of group by condition [*p* < 0.01, *F*_(2, 54)_ = 4.91, η^2^ = 0.12]. *Post-hoc* testing showed that the main effect of group was due to lower activations overall in the typical group than in the dyslexia group (*p* < 0.05). The main effect of condition was due to activations in the sentence condition being higher than in both other conditions (*p* < 0.03). The interaction effect was due to the activation for the dyslexia group in the sentence condition being higher than all other activations (*p* < 0.002). *T*-tests showed significant group differences only in the sentence condition (Typical < Dyslexia, *p* < 0.003, *d* = 1.163).

### Behavioral data

*T*-tests showed no significant differences between the groups in the number of correct responses in any of the conditions. There were no significant differences in average response times in either the alphabetic (mean dyslexia 1410.8 ms, mean typical 1248.9 ms, *p* < 0.28, *d* = 0.47) or the sentence processing (mean dyslexia 2822.0 ms, mean typical 2409.2 ms, *p* < 0.09, *d* = 0.73). There was, however, a significant difference between the groups in the orthographic condition (mean dyslexia 1849.8 ms, mean typical 1434.1 ms, *p* < 0.04, *d* = 0.90).

## Discussion

This study showed that the dyslexia group generally displayed cortical hyperactivations during reading tasks as compared to the typical group, especially in more taxing conditions. Furthermore, the difference between the groups increased with increasing processing demands. This was in line with our hypotheses. Interestingly, these group differences in cortical activation were not, with one exception, reflected in differences in performance.

Even though the different types of stimuli were generally processed in similar ways in the two groups, six cortical regions were identified where the groups responded differently to the increasing processing demands. Overall, there seemed to be a general pattern of increasing hyperactivation from the alphabetic condition to the sentence condition in the dyslexia group, and, conversely, a decrease or no change in activation in the typical group. More detailed analyses revealed that the six cortical regions could be sorted into three distinct activation patterns:

*Pattern 1* differed in the sentence condition only, where the dyslexia group showed significant increase in activation whereas the typical group showed either a decrease or no difference from the orthographic and alphabetic conditions (R-SFG-post and L-Pre-SMA).
*Pattern 2* generally showed decrease in activation in the typical group and an increase in the dyslexia group from the alphabetic to the orthographic to the sentence condition (L-NC and L-MCC).
*Pattern 3* showed decrease in activation from the alphabetic to the sentence condition in the typical group whereas the dyslexia group showed a decrease from the alphabetic to the orthographic condition and an increase from the orthographic to the sentence condition (R-SFG-ant and R-MFG).

These three patterns do not only differ in their respective BOLD responses, but may also reflect a different involvement of the underlying neuronal networks. Moreover, these networks seem to respond differently to the different processing demands, and may also respond differently to intervention.

*Pattern 1* included the right superior frontal gyrus (posterior site) and the left pre-supplementary motor area. Studies by Ni et al. (2000) and Newman et al. (2001) both found involvement of the superior frontal gyrus in sentence processing, especially in response to syntactically or semantically anomalous sentences. The pre-SMA, which is located within the superior frontal gyrus, was traditionally viewed as a motor area only. However, it has in more recent years been shown to be implicated in higher cognitive function, such as monitoring and task inhibition and switching (see Nachev et al., 2008 for a review). On the other hand, as reported earlier Richlan et al. (2009) found hyperactivation of motor areas in a number of studies, which they tied to compensatory activity. These findings were mainly from German language studies, a language that in its orthography resembles Norwegian in terms of complexity. Hence, the finding here of hyperactivation of the pre-SMA could be seen as a parallel to what Richlan et al. (2009) reported.

In pattern 1, the interaction effect was clearly driven by the sentence condition, in which the dyslexia group showed a distinct increase in activation as compared to the alphabetic and orthographic conditions. The typical group did not show this increase. For the R-SFG-post they rather showed a decrease in activation from the alphabetic and orthographic conditions to the sentence condition. There were no group differences in the first two conditions. This is largely in line with hypothesis 1 and 2. The alphabetic and orthographic conditions were expected to be relatively easy even for most of the children with dyslexia at this stage of literacy development. It is therefore not surprising that few regions show deviating activation in these conditions.

The similarities between the groups in the less complex conditions may also have been reinforced by the relatively intensive and targeted training the children underwent in the first phases of the project. Such training has previously been shown to contribute to the normalization of reading-related neuronal activity (Simos et al., 2002; Temple et al., 2003), even though further evidence may be needed. Consequently, the training may have facilitated normalized activation in the two easier conditions, whereas the sentence condition might still be challenging enough for the children with dyslexia to necessitate the recruitment of further neuronal resources in order to process stimuli, which is also in line with the studies showing that the regions in question may be involved in sentence processing and more cognitively demanding tasks (Ni et al., 2000; Newman et al., 2001; Nachev et al., 2008).

The fact that the typical group showed a decrease in activation in the sentence condition in R-SFG-post may be explained by the extra contextual information provided by the sentences. Research has shown that contextual information has a facilitative effect for both typical readers and readers with dyslexia (Assink et al., 1996). There are even indications that this facilitative effect may be more prominent in dyslexia than in typical readers (Nation and Snowling, 1998). In this view, the three conditions in question would represent three levels of contextual facilitation from low (alphabetic) to relatively high (sentences). Such facilitative factors would then interplay with the complexity of the stimuli, going the opposite way, and could do so in slightly different ways in different areas of the brain, thus explaining the three different patterns revealed in the ROIs.

*Pattern 2* was similar to Pattern 1, but also showed a significant difference between the two groups in the alphabetic condition, with the typical group displaying higher activations than the dyslexia group. The regions showing Pattern 2 were the L-MCC and the L-NC. The cingulate cortex is commonly divided into the anterior and the posterior aspects. Here, the site of activation is very much concentrated around the central region, and is as such hard to classify as either. The cingulate cortex, and especially the anterior cingulate has been implicated in a range of cognitive and emotional tasks as well as in the perception of pain and regulation of autonomic functions (see Luu and Posner, 2003 for a short review). The posterior cingulate, on the other hand, has been implicated in sentence comprehension (Cutting et al., 2006). The midcingulate cortex is, on the other hand, thought to be part of a cingulo-fronto-parietal network known to show hypofunction in ADHD (Bush, 2011), and is hence assumed to be involved in attentional mechanisms. The increased activation in the dyslexia group could thus be seen as reflecting compensatory activity. Finally, based on connectivity measures, Hoffstaedter et al. (2012) have suggested that the MCC may function as an interface between motor systems, for example the NC, and cognitive systems. Along these lines the increased activation in the NC may reflect dependency on sub-vocal articulation in reading (Richlan et al., 2009, 2011), which would necessitate some kind of connection to more cognitive areas of the brain. Furthermore, Richlan et al. (2011) suggested that hyperactivation in subcortical areas, like the caudate nucleus, may be more characteristic in younger than in older dyslexics. Similar results were reported by Wimmer et al. (2010). However, there is also evidence for more cognitive functions of the NC itself. In a review, Grahn et al. (2008) showed that the NC may be important for goal-directed action. Finally, the L-NC has been implicated in monitoring and controlling language (Tettamanti et al., 2005; Crinion et al., 2006) and more specifically in detecting syntactic anomalies (Moro et al., 2001). Overall, the dyslexia group showed the expected incremental increase in activation over the three conditions. The typical group, on the other hand, rather showed decrease in activation from condition 1 to condition 3. This could, again, show an increased dependence on cognitive resources in the dyslexia group as the processing demands are stepped up.

*Pattern 3* showed a similar decrease in activation in the typical group as Pattern 2. The dyslexia group on the other hand showed a characteristic dip in activation associated with the orthographic condition. The regions displaying this pattern were the R-SFG-ant and R-MFG. As previously mentioned, the SFG has been associated with sentence processing. As regards the MFG, Shaywitz et al. (2002) found that during a semantic category judgment task, children with dyslexia showed activation in the R-MFG not present in a group of non-impaired readers. Moreover, the MFG (bilaterally) was one of a number of regions identified by Cutting et al. (2006) as being involved in sentence comprehension. Finally, activity in the right and left MFG during phoneme judgment has been shown to correlate with activity in the inferior frontal gyrus (Richards and Berninger, 2008), a region that has been suggested to support the reading process in interpreting spoken language and providing access to semantics (Dehaene, 2009, p. 63). In our paradigm, the sentence condition was assumed to be the most complex to decode and make a semantic decision about due to the fact that it consisted of more than one single word for the children to judge. At the same time, it is also the condition that provides the highest degree of facilitative context. Hence, increased activity in regions associated with semantic processing might be expected.

Overall, the regions showing differential modulation in the two groups in response to changes in processing demand seem to be related to more general language and cognitive abilities, and may not be core to the reading network, but rather reflect compensatory activity. This is in line with a number of studies that have reported hyperactivation in dyslexia as indicative of compensatory mechanisms (for example Brunswick et al., 1999; Shaywitz and Shaywitz, 2005; Hoeft et al., 2007; Richlan et al., 2009). Right hemisphere hyperactivation has also been suggested to reflect compensation for left hemisphere hypoactivation (Démonet et al., 2004). Lehongre et al. (2011) supported this notion with the observation of compensatory activations of the right auditory cortex in dyslexics, when auditory stimuli are used that typically activate the left auditory cortex to a higher extent than the left auditory cortex, indicating a reduced sensitivity of the left auditory cortex to phonological stimuli in dyslexics. The preponderance of hyperactivation could also be a function of the language of the stimuli, and is in keeping with studies showing that in more regular orthographies the tendency is for cortical hyperactivation in groups with dyslexia (Wimmer et al., 2010). Probably, rather than being a direct consequence of language, this tendency for different activation patterns is a question of different brain mechanisms being needed to handle recoding of graphemes into phonemes in different orthographies. Also, several of the regions showing hyperactivation in the sentence condition especially are part of a network of regions connected to attention and ADHD (Bush, 2011). Importantly, none of the children in our study had ADHD. Hence, the hyperactivation in these regions could rather reflect increased engagement of an attention and cognitive network in response to more demanding stimuli. All in all, our study indicates that different types of reading stimuli may give different neuronal effects. Consequently, the type of processing demands involved in a given type of reading stimulus is important to consider when designing reading experiments. At the same time our results support the idea that the different cortical demands posed by different orthographies have consequences for the type of cortical response that could be expected.

As previously pointed out, it should be noted that the participants in this study went through three intensive training periods during the last year of kindergarten and the two first years of schooling, where literacy training started in the 2nd grade. This may have influenced the results, such that untrained children might have displayed stronger effects, seeing that targeted and evidence-based training is assumed to contribute toward a normalization of the function of the reading network (Simos et al., 2002; Temple et al., 2003). Still, both the typical and the dyslexia group received the same training (half received top-down, half bottom-up), which could have had two possible effects: (1) both groups could have benefited equally, maintaining the relative difference between the groups, but with both groups scoring better than they would have without training or (2) one group could have had more use of the training, shifting the relation between the groups. Such a group difference could go both ways; the typical group acquires literacy more effortlessly, and as such could be able to draw more benefit from the training. Conversely, the dyslexia group could have more room for improvement, and could be able to tighten the gap to the typical group by taking advantage of the targeted training. Results show that the gap in literacy between the groups widens over time, indicating that the dyslexia group is falling behind. At the same time, the gap in neurocognitive scores diminishes (Helland and Morken, in review). It is not straightforward to disentangle training effects and developmental effects in this respect. However, the dyslexia group can still be confidently diagnosed from both literacy scores and neurocognitive profiles, indicating that dyslexia has not been remediated as a result of the training sessions. Hence, we maintain that in spite of the children having received training, the results of the study are valid. We could, however, speculate that the training could have supported the development of compensatory mechanisms, contributing to the picture of hyperactivation seen in our results. As previously mentioned, there was no interaction effect between group and training scheme, indicating that the grouping into bottom-up and top-down has not affected the results significantly.

As this study has a relatively low N, strong conclusions are not warranted. Further and larger studies are needed to identify and disentangle factors of explanation, and to understand their contribution toward modulation of cortical activation in children with dyslexia in more detail. Also, due to the low N, further subdivision of the groups (for example into subtypes of dyslexia) is not supported, even though this could be of interest in a larger sample.

This study indicates that changes in literacy processing demand results in different cortical activation patterns in children with and without dyslexia. In general, the differences seem to increase in magnitude as the tasks get more demanding, which is also in line with the developmental nature of dyslexia and of reading as a skill that develops over time. The observed hyperactivations are suggested to be chiefly associated with compensatory mechanisms and attention, possibly reinforced by the evidence-based training provided to the children in the first years of the project. However, the differences do not seem to be explainable from stimulus complexity alone. We have suggested facilitative contextual clues as one modifying factor. Finally, our study shows that when designing studies of cortical activation in dyslexia, it is important to consider the type of processing demands posed by the stimuli chosen, and that using stimuli of different complexity may lead to broader insight into the nature of this disorder.

### Conflict of interest statement

The authors declare that the research was conducted in the absence of any commercial or financial relationships that could be construed as a potential conflict of interest.
